# Isolated Carpometacarpal Dislocation Without Associated Fracture: A Case Report of High-Energy Wrist Trauma

**DOI:** 10.7759/cureus.92461

**Published:** 2025-09-16

**Authors:** Mohammed S Al Saleem, Fatimah ALthabit, Arwa H AlOnayzan, Khadijah Alruwaili, Adia Almutairi

**Affiliations:** 1 Orthopedic Surgery, King Fahad Hospital, Hofuf, SAU; 2 Orthopedics, King Fahad Hospital, Hofuf, SAU; 3 Orthopedic Surgery, King Fahad Military Medical Complex (KFMMC), Dammam, SAU; 4 Orthopedics, King Abdulaziz Hospital, Al-Mubarraz, SAU

**Keywords:** carpometacarpal dislocation, hand surgery, high-energy injury, k-wire fixation, wrist trauma

## Abstract

Carpometacarpal (CMC) joint dislocations without fractures are rare and often missed due to subtle clinical and radiographic signs. We report a case of a 28-year-old male patient involved in a motorbike rollover who sustained open comminuted radius and ulna fractures, an ulnar styloid fracture, and dorsal dislocation of multiple CMC joints without metacarpal fractures. He presented with absent distal pulses and finger numbness, which resolved after emergency reduction. CT angiography ruled out arterial injury. He underwent internal fixation of forearm fractures and Kirschner wire stabilization of CMC joints. At four months, he achieved full wrist and finger motion, normal grip strength, and no instability. Early diagnosis and timely surgical intervention were key to recovery.

## Introduction

Isolated dislocations of the carpometacarpal (CMC) joints are uncommon, comprising less than 1% of all hand and wrist injuries [1]. They are typically the result of high-energy trauma, such as motor vehicle accidents or falls from significant height. Among these, dislocations of the ulnar-side joints, particularly the fourth and fifth CMC joints, are most frequent, often occurring dorsally due to the strong volar ligamentous support [2]. These injuries are frequently overlooked in the acute setting, especially when accompanied by more apparent or urgent trauma, as clinical signs may be subtle and conventional wrist radiographs can fail to detect the dislocation due to overlapping osseous anatomy [1,2]

Accurate and timely diagnosis requires a high index of suspicion, supplemented by appropriate imaging. While standard dorsopalmar, lateral, and oblique radiographs are essential for initial assessment, computed tomography (CT) remains the gold standard for confirming dislocations, identifying subtle fractures, and aiding surgical planning [3]. Missed or delayed diagnosis may lead to poor outcomes, including chronic pain, grip weakness, joint instability, tendon irritation, and early-onset post-traumatic arthritis [2,3].

Management depends on the severity and timing of the injury. While acute, isolated CMC dislocations may be treated with closed reduction, post-reduction instability is common, often requiring percutaneous Kirschner wire (K-wire) stabilization [4]. In complex injuries, such as open dislocations, those with associated fractures, or delayed presentations, open reduction and internal fixation (ORIF) is typically indicated to restore anatomical alignment, decompress neurovascular and soft tissue structures, and enable early mobilization [5].

The purpose of this study is to highlight a rare and clinically important case of isolated CMC dislocation without metacarpal fractures, combined with open forearm fractures in a young man following high-energy trauma. This case underscores the need for early recognition, advanced imaging, and timely surgical management, contributing valuable insight to the limited literature on complex CMC injuries.

## Case presentation

Clinical presentation and history

A 28-year-old medically free male was presented to the Emergency Department (ED) after a high-energy road traffic accident (RTA) involving a motorbike rollover. Upon arrival, he was conscious, alert, and fully oriented with a Glasgow Coma Scale (GCS) score of 15/15. He was hemodynamically stable, with a temperature of 37°C, a heart rate of 74 beats per minute, a respiratory rate of 22 breaths per minute, a blood pressure of 119/72 mmHg, and an oxygen saturation of 98% on room air.

Primary assessment confirmed an intact airway and spontaneous breathing. A large-bore intravenous cannula was inserted, and 500 mL of normal saline was administered after blood was sent for initial laboratory investigations. No signs of intra-abdominal injury were noted, and the pelvis was clinically stable.

Examination

**Figure 1:**
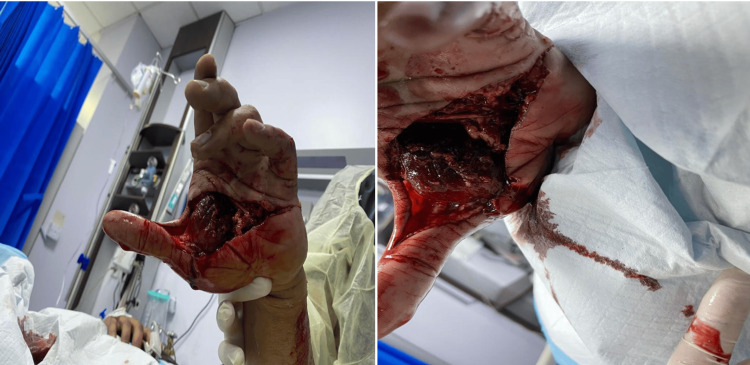
Open volar wound with severe swelling and deformity of the hand on presentation

Focused examination of the left forearm revealed an open, comminuted fracture with severe swelling and a visible wound on the volar aspect of the hand (Figure 1). The patient reported numbness in all fingers. Pre-reduction assessment showed absent distal radial and ulnar pulses. After gentle alignment in the ED, pulses remained impalpable but were confirmed by Doppler ultrasound.

Investigations and procedure

**Figure 2:**
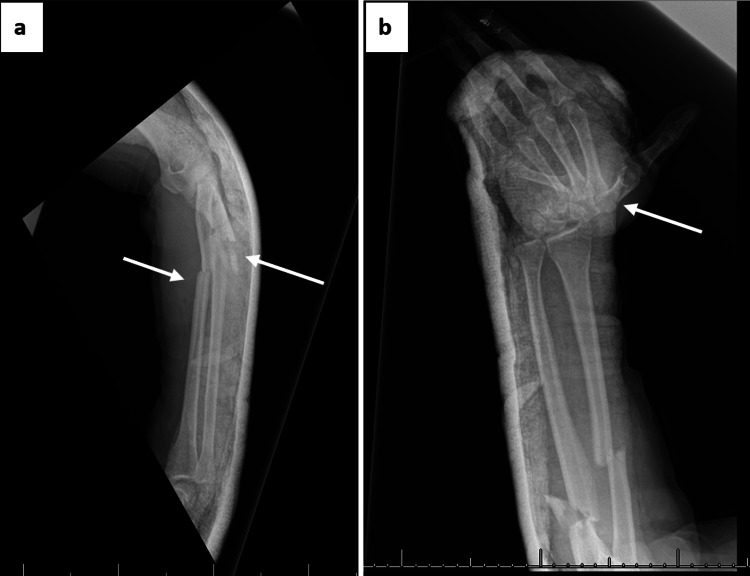
Initial radiographs: (a) lateral view showing comminuted fractures of the radius and ulna, (b) anteroposterior view demonstrating ulnar styloid fracture and wrist dislocation.

**Figure 3:**
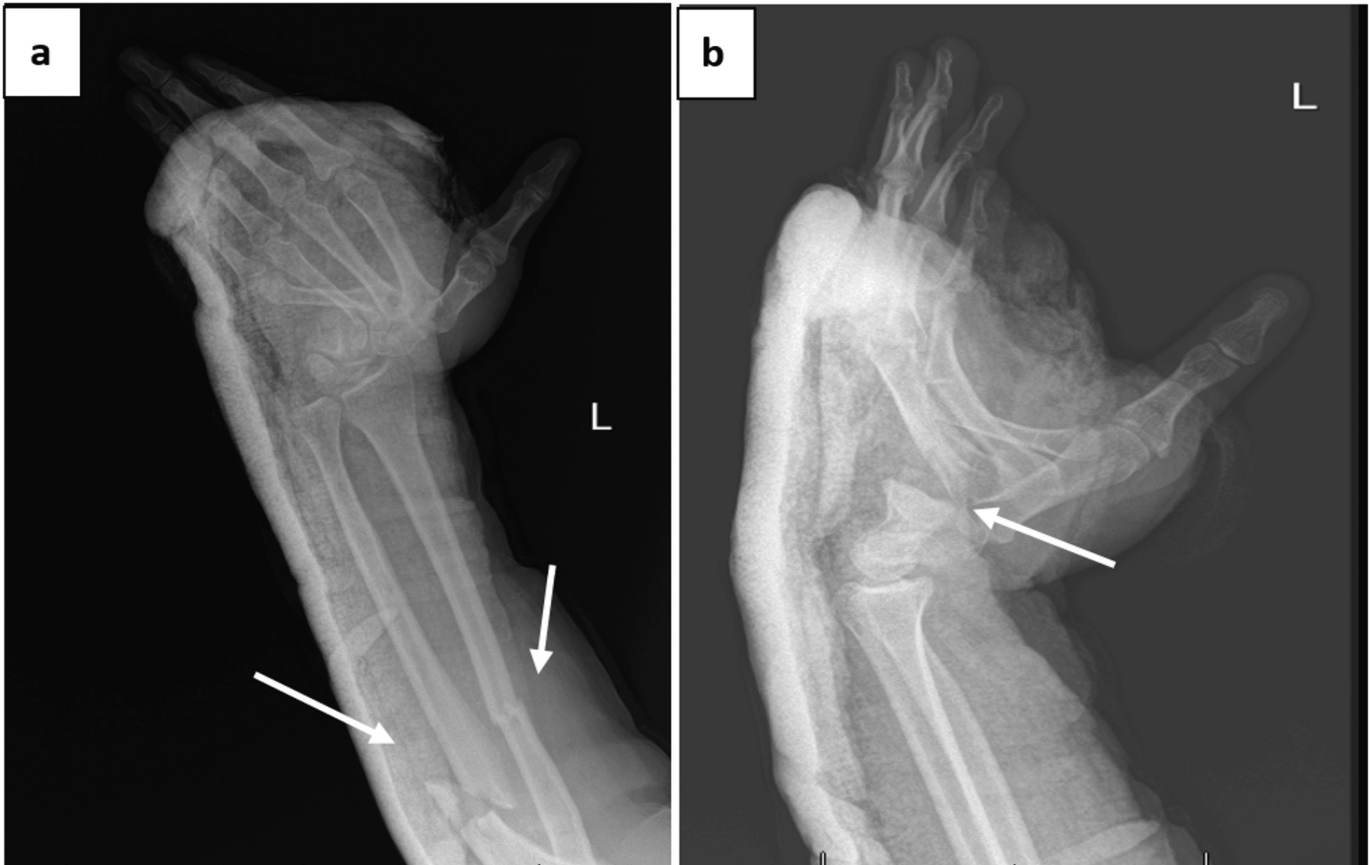
Additional X-ray views confirming left carpometacarpal (CMC) joint dislocation: (a) oblique view, (b) lateral view highlighting dorsal displacement

Initial radiographs revealed diaphyseal comminuted fractures of the radius and ulna, an ulnar styloid fracture, and dorsal dislocation of the wrist joint (Figure 2). Additional views confirmed the presence of a dorsal carpometacarpal dislocation (Figure 3).

A CT angiogram demonstrated intact subclavian, axillary, and brachial arteries. The radial artery showed segmental attenuation without a cutoff; the ulnar artery was intact. Vascular surgery consultation concluded that no intervention was needed due to the absence of major arterial injury.

**Figure 4:**
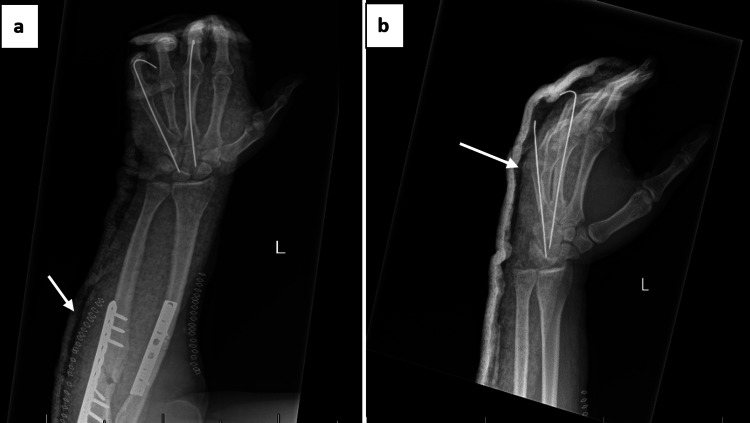
Intraoperative images: (a) ORIF of radius and ulna using DCP, (b) K-wire fixation of carpometacarpal joints. ORIF: open reduction and internal fixation; DCP: dynamic compression plate; K-wire: Kirschner wire

**Figure 5:**
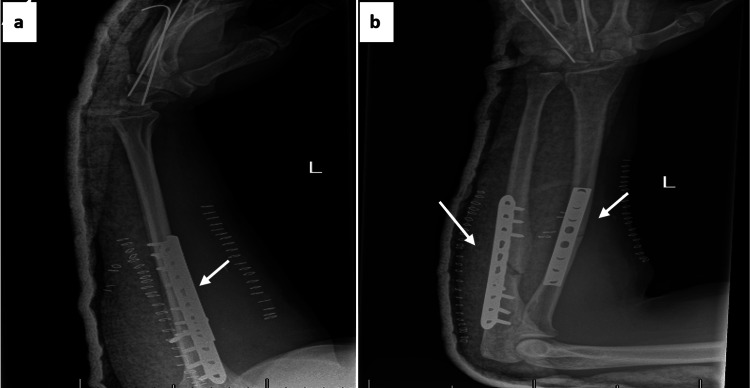
Postoperative radiographs: (a) lateral view showing stable fixation of forearm fractures, (b) anteroposterior view confirming anatomical reduction and implant position

The patient underwent urgent ORIF of both radius and ulna using dynamic compression plates, along with percutaneous K-wire fixation of the dislocated carpometacarpal joints for stabilization (Figure 4). Final intraoperative X-rays confirmed appropriate reduction and hardware positioning (Figure 5).

Postoperative management and follow-up

**Figure 6:**
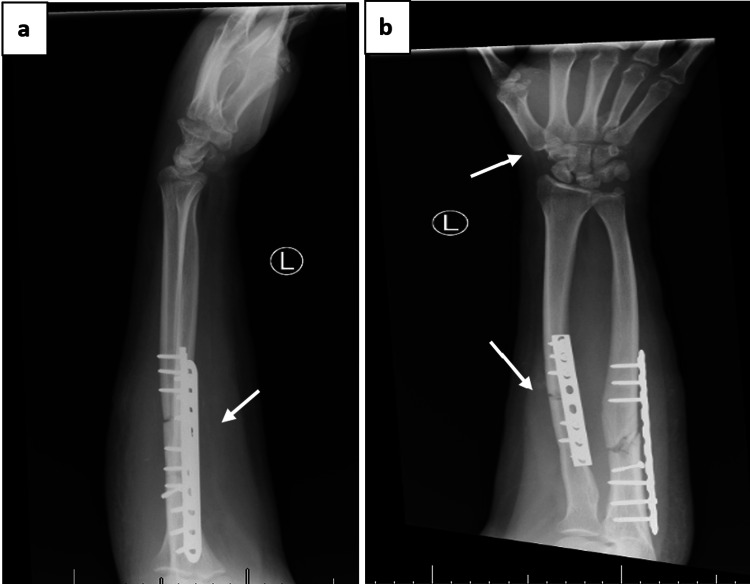
One-month follow-up after removal of K-wires: (a) lateral view showing stable fixation of radius and ulna with plates and screws, (b) Anteroposterior view confirming maintained alignment and absence of K-wires

The patient was monitored postoperatively for neurovascular status, wound condition, and signs of infection. Pain management and intravenous antibiotics were administered according to hospital protocol. One month after surgery, the K-wires were removed, and the patient began a structured physiotherapy program focusing on wrist mobility and grip strength restoration. Post-K-wire removal radiographs are shown in Figure 6.

**Figure 7:**
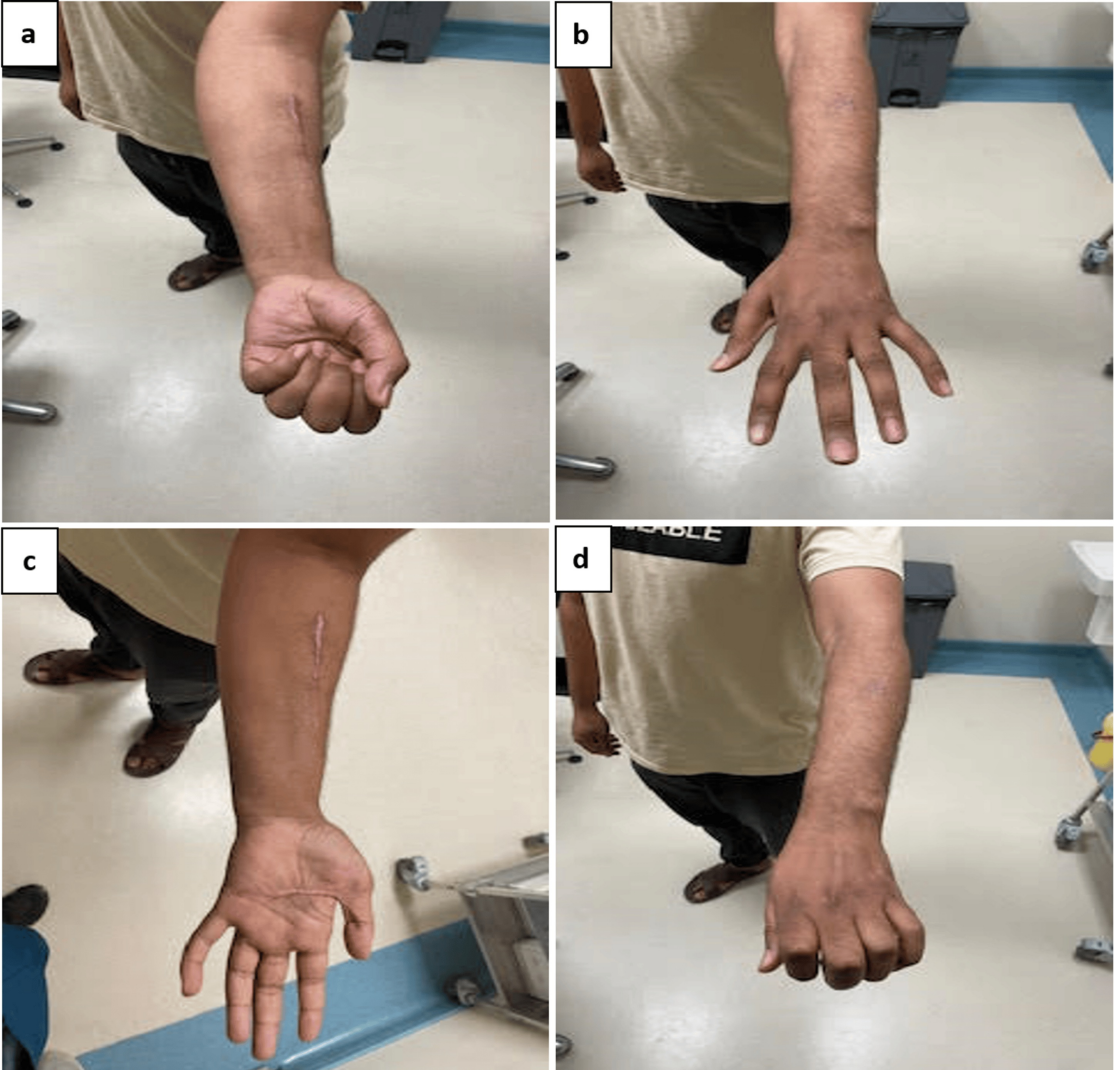
Clinical photos at four months demonstrating full recovery of wrist motion and hand function: (a) wrist flexion, (b) wrist extension, (c) forearm supination, (d) forearm pronation

At the four-month follow-up, the patient demonstrated a full range of motion in the wrist and fingers, normal grip strength, and no evidence of residual instability or pain. He was able to return to normal daily activities with no functional limitations. Clinical recovery is shown in Figure 7.

## Discussion

CMC joint dislocations are rare, accounting for less than 1% of all hand and wrist trauma cases [3]. Diagnosis is often delayed due to subtle clinical signs and the overlapping anatomy of carpal and metacarpal bases, which can obscure findings on standard radiographs [3]. In the present case, the diagnosis was not immediately apparent from initial plain films due to complex forearm fractures and extensive soft tissue swelling. However, a high index of clinical suspicion, combined with advanced imaging, confirmed the dorsal dislocation of multiple CMC joints. This supports current recommendations for obtaining three-view radiographs, dorsopalmar, lateral, and oblique, and utilizing CT when findings remain equivocal [6].

Management of CMC dislocations is influenced by the timing of presentation and joint stability after reduction. While closed reduction may be successful in early, uncomplicated dislocations, open reduction is often required in cases of open injuries, fracture-dislocations, or delayed presentation due to interposed soft tissue, bony fragments, or severe edema [7]. In this report, the patient presented acutely with open comminuted fractures of the radius and ulna, along with CMC dislocation, necessitating open surgical intervention to achieve adequate reduction and stabilization.

Post-reduction instability is common in complex CMC dislocations and frequently requires temporary internal fixation. In this case, the extent of injury and associated soft tissue damage resulted in instability following reduction, which was managed with percutaneous K-wire fixation to maintain anatomical alignment. This approach is consistent with evidence suggesting that K-wire stabilization is indicated when joint congruity cannot be reliably maintained by closed means [8]. This case also contrasts with reports of isolated CMC dislocations successfully treated with splint immobilization [9], highlighting the need to individualize treatment based on injury complexity.

The mechanism of injury in this case, a high-energy motorbike rollover, is relatively uncommon in published series. A large retrospective study involving 4,751 patients reported punching as the most common cause of CMC dislocation (73%), followed by blunt trauma (18%) and road traffic accidents (9%) [9]. Additionally, several patients in that study were misdiagnosed initially and presented in a delayed fashion. In contrast, early recognition and coordinated surgical management in the present case may have contributed to the favorable functional outcome.

Despite the potential for complications such as tendon adhesions, stiffness, post-traumatic arthritis, and complex regional pain syndrome, many patients recover well following prompt intervention and structured rehabilitation [10,11]. In the current case, the patient regained full wrist and hand function by four months postoperatively, with no residual pain, weakness, or instability. This outcome is consistent with previous reports demonstrating excellent functional recovery when anatomical reduction is achieved and early rehabilitation is implemented [12].

## Conclusions

Isolated CMC dislocation without metacarpal fracture is a rare and easily missed injury. This case highlights the value of early recognition, advanced imaging, and timely surgical management. ORIF and K-wire stabilization resulted in excellent functional recovery, emphasizing the importance of individualized treatment in complex hand trauma.
